# Editorial: Tokyo 2020 Olympic and Paralympic games: Specificities, novelties and lessons learned

**DOI:** 10.3389/fspor.2022.1026769

**Published:** 2022-09-16

**Authors:** Grégoire P. Millet, Yuri Hosokawa, Øyvind Sandbakk, Olivier Girard

**Affiliations:** ^1^Institute of Sport Sciences, University of Lausanne, Lausanne, Switzerland; ^2^Faculty of Sport Sciences, Waseda University, Tokorozawa, Japan; ^3^Department of Neuromedicine and Movement Science, Centre for Elite Sports Research, Norwegian University of Science and Technology, Trondheim, Norway; ^4^School of Human Science (Exercise and Sport Science), The University of Western Australia, Crawley, WA, Australia

**Keywords:** Tokyo 2020 Olympic and Paralympic games, periodization, strength training, heat, COVID-19

The Tokyo 2020 Summer Olympics (23 July−8 August 2021) and Paralympic Games (24 August−5 September 2021) posed unique challenges: First, they were postponed by 1 year; Secondly, the COVID-19 pandemic was not over, and consequently specific measures were taken prior and during the competitions; Thirdly, competitions were held in the hottest environment in the history of the games.

These three points may have impacted competition outcomes for the following reasons:

1. Due to the worldwide COVID-19 pandemic, events originally scheduled for 2020 were postponed to 2021. This situation is unique in the Olympic and Paralympic history and required considerable preparation adjustments for the event organizers, the athletes and their supporting teams.
2. The training regimen, preparation and selection of the athletes as well as the antidoping tests were perturbed by national lockdown policies, which differed considerably between countries.
3. In many sports, no spectators were permitted, creating an “experimental set-up” to evaluate the influence of the crowd on athletes' performance in major competition.
4. For safety reasons, the duration of stay in Japan and/or in the Olympic/Paralympic village was restricted when compared to previous Olympics and Paralympics. This was an additional challenge to recovery from travel fatigue and/or jet lag and for acclimatization to local environmental conditions for many teams who normally live and/or compete in European or American countries.
5. Similarly, the travel bans leading up to the Olympic and Paralympic Games and the COVID-19 related restrictions after entering Japan have significantly impacted team's original plans of training regimen leading up to the Games.
6. Important biosecurity protocols were implemented such as practicing social distancing, maintaining high hygiene standards, and wearing face masks. This may have had detrimental effects on athletes' wellbeing.

Two articles published in this Research Topic focused on preparation and periodization.

Rønnestad presented a case study with elite cyclists periodizing heavy strength training during multiple training and competition periods. Muscle strength improvement was observed during preparatory period, as expected, but was also maintained over several years.

van der Zwaard et al. investigated changes in performance and in muscle morphology in elite female rowers. The authors showed that *vastus lateralis* muscle adaptations differed largely between individual athletes and was highly adaptive to the specific training stimuli. Accordingly, assessing athletes' individual (muscle) adaptations is key to evaluate training adaptations and adapt training stimulus individually.

Four articles investigated how the pandemic situation modified the preparation of the athletes and/or the delivery of medical services.

Lundqvist and Kristiansen compared perceived challenges along with risk and protective factors for mental health in athletes from Norway (affected by lockdown) and from Sweden (not/less affected by lockdown) in preparation to the Olympics Games. Several coping strategies were discussed.

Muti et al. described the successful implementation of a 10-month protocol in Italian para rowing-athletes prior the Paralympic Games. The results indicated that developing an anti-COVID-19 protection “bubble” is feasible, which could be extended to athletes engaged in other sports for infection protection.

Nakamura et al. reported how heat protective measures, including heat acclimation and cooling strategies, were implemented by the 3x3 Japan national basketball team, and how protective measures against COVID-19 were considered.

Finally, Sugawara et al. detailed strategies used by medical teams to keep athletes cool available at the marathon and race walking Olympic events. During these events, which were located to Sapporo, the incidence of exertional heat illness was prevalent in most athletes who were admitted to the athlete medical station.

In addition to the challenges associated with organizing a major competition in hot and coronavirus environments, specificities and novelties at the summer 2020 Olympic and Paralympic Games came from the changes implemented in the sporting programs:

1. New sports (karate, sport climbing, surfing and skateboarding) and new event formats (e.g., 3x3 basketball, freestyle BMX, and several mixed gender teams) were introduced in the Olympic program. Scientific investigations of these mixed gender events are still lacking.
2. Badminton and taekwondo were added to the Paralympic program but are basically unexplored scientifically.
3. Several preventive measures have been endorsed by organizers to beat the heat and to protect athletes' health during summer months in Tokyo. While many of these appeared to work well for practice, understanding of the underlying mechanisms are unknown.
4. Many world records were broken in swimming, track cycling and athletics. The exact reasons for improved performances may vary between sports and, in many cases, remain to be better understood.
5. A total of 93 countries/regions won at least one medal, which is the highest ever achieved, and shows a continuous increasing globalization of the Olympic movement. Seventy-eight countries won at least one medal during the Paralympic games, which is slightly less than the 83 countries achieving a medal in the 2016 Paralympic games.

Important changes in the medal tables were observed (e.g., The Netherlands and Italy entering in the top-10 medal table of the Olympic games). Considering the Paralympic Games, the top medaling countries remained similar (e.g., China, Great Britain, United States, and Ukraine).

Two articles of the present Research Topic investigated the factors influencing the medals/nations ranking and proposed new models for measuring nations or medals tallies:

Li et al. proposed a model adjusting the medal tallies in both Olympics and Paralympic games for demographic and geographic factors. Authors argued that such comparison is more in keeping with the Olympic ideal and is also more useful for nations that base their Olympic-funding decisions on medal performance.

Li and Hopkins further proposed to modify the rules for increased shared medal winning, based on the score variability between sports.

To conclude, this Research Topic covered mainly three Research Topics: athletes' preparation; measures against COVID-19; and factors influencing medal ranking of nations. Some of the lessons learned from these studies, and practical experience in the Tokyo 2020 Summer Olympics and Paralympic Games would need to be considered by future host countries (e.g., Paris 2024 Olympics, Los Angeles 2028 Olympics) to protect/maintain athletes' health and develop performance further. In addition, such major events provide us as sport scientists not only with inspiration but also generate new, testable hypotheses that would advance our field further!

## Author contributions

All authors listed have made a substantial, direct, and intellectual contribution to the work and approved it for publication.

## Conflict of interest

The authors declare that the research was conducted in the absence of any commercial or financial relationships that could be construed as a potential conflict of interest.

## Publisher's note

All claims expressed in this article are solely those of the authors and do not necessarily represent those of their affiliated organizations, or those of the publisher, the editors and the reviewers. Any product that may be evaluated in this article, or claim that may be made by its manufacturer, is not guaranteed or endorsed by the publisher.

